# 

**Published:** 1879-02

**Authors:** 


					ARTICLE IX.
Baltimore College of Dental Surgery.
?
The Thirty-Ninth Annual Commencement of this College
will be held in the Academy of Music, Baltimore, on the
evening of March 6th, 1S79. The Valedictory Address
will be delivered by Rev. Alex. W. Weddell, D. D., of
Richmond, Ya. The Class Address will be delivered by
Frank B. Perry, of Bridgewater, Ya.
The Alumni and all friends of the Institution are coi-
dially invited to attend. Stage and other tickets can be
obtained of the Dean, and all attending will be cordially
welcomed.
Alumni Meeting?Baltimore College of Dental Surgery.
The Annual Meeting of the Alumni Association will be
held in the College Bnilding, March 6th, 1879, commencing
at 10 A. M. As the Annual Meeting for 1879 promises to
be a very interesting one, all the Graduates of this Colleeg
Alumni lie-Union of Ohio Dental College. 469
are earnestly requested to be present. The present pros-
perous condition of this, the oldest of Dental Colleges, is a
matter for congratulation to all its Alumni who are inter-
ested in dental education.
S. J. CoCKERILLE, D. D. S.,
Pres. A lurnni A ssociation.
Wm. B. Wise, D. D. S., Thos. H. Davy, D. D. S.,
Corresponding Secretary. Recording Secretary.
Mississippi Valley Dental Association.
The Thirty-Fifth (35th) Annual Meeting of the Mississippi
Valley Dental Association will take place in Cincinnati,
O., in Dental College Hall, on the first Tuesday in March,
1879, at 10 o'clock, A. M.
President ?Frank A. Hunter.
lstf Vice President.?J. S. Cassidy.
2nd Vice President.?J. W. Jay.
Recording Secretary.?E. G. Betty.
Corresponding Secretary.?C. I. Keely.
Treasurer.?J. G. Cameron.
E. G. Betty,
Secretary.
First Alumni Re- JJnion of Ohio Dental College.
At the close of the last session of the Ohio College of
Dental Surgery, it was decided by those interested who
were present, to hold a re-union of all the Alumni at the
time of the next annual commencement, when it is proposed
to have every class that has ever gone out from the Institu-
tion as fully represented as possible. The object of the
re-union is to bring together the graduates of the college
upon the Ter-centenary Anniversary, that they may revive
acquaintance and pledge themselves anew to give her that
support and aid which enhances the usefulness of educa-
cational institutions.
A general committee consisting of one member from each
class was appointed, whose duty it shall be to interest their
470 American Journal of Dental Science.
respective classes in the meeting, and to furnish pithy
biographical sketches of deceased members. They will also
give the leading items of interest pertaining to the classes,
present location, status, etc., of the members, the reports to
be made in the order of graduation.
The executive committee appointed, will attend to all
arrangements necessary for the success of the meeting and
entertainment of those attending. It is earnestly desired
that every one will respond to this call, and by his presence
give assurance of continued interest in his time-honored
Alma Mater.
Programme.?Thursday, March 6th, 1879, 2 P. M.
Organization of meeting. Calling the roll. Miscellaneous
business. Historical Address, Dr. Geo. Watt, Class 1854.
Reading of reports from General Committees in the order
of graduation. The evening to be devoted to an entertain-
ment, sentiments, toasts, etc.
Effort is being made by the Faculty to extend and render
attractive the Museum of the College. The co-operation of
the Alumni is earnestly desired in this work.
Donations of Specimens and Models of Anomalous Cases
are respectfully solicited. Send to H. A. Smith, Dean. 286
Race St., when suitable acknowledgement and thanks for
the same will be made.
Executive Committee.?J. Taft, Chairman / J. S. Cassidy,
H. A. Smith. E. G. Betty, Secretary.
To Readers and Correspondents.
Communications intended for publication, and exchange journals and papers,
should be addressed to the Editor of the American Journal of Dental
Science, 259 N. Eutaw St., Baltimore, Md. All letters relating to the business
of the journal, or containing cash remittances, to be directed to Messrs. Snowden
& Cowman. Articles for publication should be received by the editor at least
three weeks previous to the first day of the month on which the number in which
it is designed for them to appear, is issued.
I^^The American Journal of Dental Science will contain fifty pages of
reading matter in each number, making a volume of about
Six Hundred pages
EXCLUSIVE OF ADVERTISEMENTS
T H K
L
iMEBICANJOURNAL
OF
DENTAL SCIENCE.
The Journal is issued on the first of each month and contains not less
than fifty pages of reading matter in each number, exclusive of adver-
tisements, making a volume of six hundred pages per annum.
In each number will be found Original Communications. Corres-
pondence, Selected Articles, Monthly Summary, Bibliographical No-
tices and Editorial Matter, and every effort will be exerted to render
the Journal worthy 01 the patronage of the Dental profession.
A generous co-operation is respectfully solicited from the members
oi the profession; and as the Journal has now a printing office of its
own, which materially lessens the cost of its publication, it will be
issued at the reduced subscription price of
I.SO Per Annum ?xx Advance.
Communications are respectfully solicited on all subjects pertain-
ing to the practice of dentistry, as well as reports of cases occurring
in dental practice.
RATES OF ADVERTISING.
I Page.
* "
i "
i ?
1 MO.
$15 00
10 00
7 00
5 00
2 MO.
$22 50
15 00
11 00
8 00
3 MO.
$30 00
20 00
15 00
11 00
6 MO.
$50 00
30 00
20 00
15 00
12 MO.
$100 00
50 00
30 00
20 00
All original communications designed for publication, works for
review, new instruments for notice, exchanges, &c., should be directed
to the editor, 259 N. Eutaw St., Baltimore, Md.
All advertisements and subscriptions should be addressed to
SNOWDEN & COWMAN,
Publishers of the Am. Journal of Dental Science.
86 W. Fayette St. Bet. Charles & Liberty Sts.,
BALTIMORE, MD

				

